# In Vitro Models of Bacterial Biofilms: Innovative Tools to Improve Understanding and Treatment of Infections

**DOI:** 10.3390/nano13050904

**Published:** 2023-02-27

**Authors:** G. Crivello, L. Fracchia, G. Ciardelli, M. Boffito, C. Mattu

**Affiliations:** 1 Department of Mechanical and Aerospace Engineering, Politecnico di Torino, C.so Duca degli Abruzzi 24, 10129 Torino, Italy; 2 Department of Pharmaceutical Sciences, Università del Piemonte Orientale “A. Avogadro”, Largo Donegani 2, 28100 Novara, Italy; 3 Department of Life Sciences, University of Modena and Reggio Emilia, Via Campi 287, 41125 Modena, Italy

**Keywords:** biofilm models, infection, bacteria, 3D printing, microfluidics, microcosm models, in vitro models

## Abstract

Bacterial infections are a growing concern to the health care systems. Bacteria in the human body are often found embedded in a dense 3D structure, the biofilm, which makes their eradication even more challenging. Indeed, bacteria in biofilm are protected from external hazards and are more prone to develop antibiotic resistance. Moreover, biofilms are highly heterogeneous, with properties dependent on the bacteria species, the anatomic localization, and the nutrient/flow conditions. Therefore, antibiotic screening and testing would strongly benefit from reliable *in vitro* models of bacterial biofilms. This review article summarizes the main features of biofilms, with particular focus on parameters affecting biofilm composition and mechanical properties. Moreover, a thorough overview of the in vitro biofilm models recently developed is presented, focusing on both traditional and advanced approaches. Static, dynamic, and microcosm models are described, and their main features, advantages, and disadvantages are compared and discussed.

## 1. Introduction

Bacteria are versatile microorganisms that can easily adapt to different environmental conditions through the modulation of their genetic expression, in particular by turning on stress-response genes [1]. For instance, bacteria can tune their growth rate according to nutrient and oxygen availability [2] and adapt to survive under extremely harsh conditions, such as the low pH conditions of the gut [3] or the high temperatures of hot springs [4].

One of the main strategies to survive harsh environments is to shift from an individual and proliferative state (planktonic state) to a more organized and static condition (sessile state) [5]. In the sessile state, bacteria are packed in complex communities embedded in an extracellular matrix (ECM) known as biofilm, which is composed of a wide range of macromolecules produced by bacteria called extracellular polymeric substances (EPS). EPS, which are mainly exopolysaccharides, proteins, and DNA, form three-dimensional structures that improve intercellular communications and nutrient exchange, while also providing protective functions [5,6].

Bacteria embedded in biofilms are more resistant to chemical challenges, such as exposure to disinfectants and antibiotics [5,7]. This is particularly relevant in the medical field since more than 80% of the infections that develop in the body are associated with biofilm formation [5,8]. Biofilm-associated infections can develop spontaneously in different organs and tissues, or they may arise after the implantation of external devices [9]. In the first case, the patient often develops chronic infections, such as cystic fibrosis, osteomyelitis and non-healing wounds [5,10]. In the second case, the infection begins on and develops from the surface of implants such as heart valves, orthopedic prostheses and catheters [11]. For example, *Staphylococcus aureus* and *Staphylococcus epidermidis* can form biofilms on the surface of bone fixation devices, which result in difficult to eradicate acute or chronic osteomyelitis [12]. Similarly, urinary catheters are prone to infection by various microorganisms, especially *Escherichia coli*. In this case, fibrinogen or other constituents of the urine form a film on the catheter surface which supports bacterial adhesion and biofilm formation [13].

Because of their chronic nature and heterogeneity, biofilm-associated infections are a serious threat for the patients and a burden for the health care system. Moreover, the spread of antibiotic resistance has reduced the effectiveness of the available treatments, requiring the development of innovative and more effective strategies. In 2017, multidrug-resistant bacterial infections affected more than 350 thousand people in the US costing around USD 1.9 billion [14].

Reliable models that mimic the complexity of the biological environment have the potential to advance our knowledge on the biofilm dynamic and composition and to improve the screening and the design of new treatment options. Unfortunately, the biofilm architecture as well as the specific EPS composition are strongly influenced by the bacterial species involved and by the environmental conditions, making biofilm extremely variable and complex to understand and model [6,15,16,17,18]. Animal and *in vitro* models of infections have been widely used [5,19,20]. However, traditional 2D *in vitro* models tend to be simple and unable to reproduce the complexity found in nature, while *in vivo* models are expensive and raise ethical concerns [21]. In the last decades, increasing attention was put on developing complex *in vitro* 3D models of biofilms using innovative technologies. For instance, 3D-printing technology allowed a more accurate reproduction of the biofilm architecture [22], while microfluidic devices have been exploited to mimic specific environmental conditions, such as flow or nutrient gradients [23,24]. Moreover, these models can include human cells as well as ECM components or can be integrated with other tissue models, with the aim to better mimic the interactions between bacteria and the host [25,26]. These systems are known as microcosm models.

The aim of this review is to present the latest advances in biofilm modelling. A brief introduction will guide the reader through the current understanding of biofilms and the challenges associated with their modelling. Then the most promising models will be discussed, and the main unsolved issues will be highlighted.

## 2. Lifecycle and Structure of Biofilms

Biofilms are complex communities in which bacteria of different species cooperate. Their architecture and their mechanical and chemical properties depend on several factors, mainly related to the types of bacteria present and the environmental conditions under which the biofilm grows [5]. The biofilm lifecycle is complex, and a variety of processes occur and overlap during its development, depending on the bacterial species involved and on the characteristics of the substrate.

**Figure 1:**
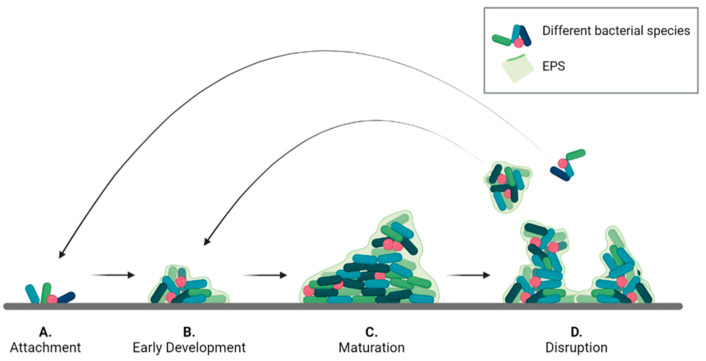
Bacterial biofilm life cycle divided into the four phases: (**A**) attachment, (**B**) early development, (**C**) maturation, and (**D**) disruption.

A common development pattern can be identified for all types of biofilms, divided into the four phases described in Figure 1 [17,20,27]. Initially, single planktonic cells and/or aggregates attach to the surface by transient interactions that later develop into a more robust adhesion. The success of this phase, known as attachment (Figure 1A), depends on different factors including surface chemistry and morphology, temperature, pressure, presence of flow, as well as steric or electrostatic interactions [20,28]. For instance, *S. epidermidis* has been shown to colonize titanium implant surfaces to a different extent depending on their roughness [29]. Moreover, some bacteria present filamentous structures, such as pili and flagella, that can interact with surfaces to promote adhesion [30,31,32]. Once the bacteria are stably adherent, surface colonization begins by producing EPS [33]. In the early development stage (Figure 1B), bacteria divide and recruit other planktonic cells by aggregation and agglutination [5,34]. Later, bacteria expand to form microcolonies by chemically communicating with each other and by expressing genes for EPS secretion [35,36]. In this phase, a 3D architecture is developed to allow network stabilization and to facilitate intercellular signaling [37].

In the maturation phase (Figure 1C) the bacterial aggregates thicken and become macro-colonies [38]. In this process, that may take several days, gradients of nutrients, oxygen and pH within the biofilm, play a pivotal role influencing bacterial metabolism in the different biofilm regions [39,40,41]. As a result, a variety of biofilm architectures can be observed depending on bacterial species, EPS composition and environmental conditions [6,15]. As an example, *Pseudomonas aeruginosa* biofilms can form a mushroom-shaped structure when glucose is used as carbon source, or a flat and densely packed structure in the presence of citrate [42]. Flat-shaped biofilms are often observed *in vitro* across different bacterial species, such as *Frascinella novicida* [43], *Staphylococcus xylosus* and *S. aureus* [44]. However, biofilms formed *in vivo* have more variegated shapes. For instance, Kim et al. [45] observed four different biofilm shapes in polymicrobial infections of teeth obtained from patients. The different shapes reflected different compositions of bacterial species. Moreover, floating aggregates can also form in the presence of mucosa bacteria. This is the case of biofilms formed in lungs and wound beds [46].

After reaching maturity, biofilms undergo a partial disruption (Figure 1D) that is mediated by two different processes: detachment, and dispersion. In the first case, external forces, such as mechanical and shear stress, cause the release of biofilm portions. The released cells are in a sessile state and are partially embedded in the EPS matrix, which protects them from the environment [15,17]. Dispersion is a response to internal stress, such as high proliferation rate, or to external stimuli, such as variation in nitric oxide or oxygen levels [47,48]. These stimuli induce cells to switch back en masse to the planktonic state and to escape the biofilm to colonize other areas. The result is the formation of voids or cavities within the biofilm structure. Moreover, the process leaves the released cell more vulnerable to external chemicals, such as antibiotics and disinfectants [48].

Biofilm architecture and composition are highly heterogeneous with clusters of cells incorporated in EPS and interstitial voids that facilitate oxygen and nutrient transport [6,49,50]. The cells within a biofilm can have different phenotypes because of the gradients of nutrients and oxygen. For instance, bacteria in the inner part of biofilm have lower metabolic activity, since their access to nutrients is limited, and are more resistant to antimicrobial agents. These cells are known as ‘*persister cells*’, having a dormant phenotype that can switch back to an active state under favorable environmental conditions [5,51].

### 2.1. The EPS Composition

The EPS matrix may account for 50% to 90% of the biofilm composition, depending on the bacterial strains present, the stage of biofilm development and the environmental conditions (e.g., shear forces, temperature, and nutrient availability) [5]. These factors also influence the composition of the EPS, giving rise to a multitude of different biofilms [5,6]. As stated above, the EPS is composed mainly of exopolysaccharides, proteins and extracellular DNA (eDNA) [6,7,16].

Exopolysaccharides are the major fraction of the EPS and are essential for biofilm maturation since they are involved in nutrient sequestration and biofilm attachment. Exopolysaccharides are long-chain polymers mainly composed of carbohydrates. Each bacterial species assembles specific exopolysaccharides. For example, alginate, a polyanionic exopolysaccharide composed of uronic acid, is characteristic of *P. aeruginosa* biofilms [52,53]. On the contrary, in *S. aureus* infections, biofilms are composed of polysaccharide intercellular adhesin (PIA), which is a polycationic exopolysaccharide [54]. Dong et al. showed that cationic liposomes penetrate deeper and in higher quantity in *P. aeruginosa* biofilm compared to anionic liposomes, confirming that the biofilm net charge is another fundamental aspect to consider when designing treatment strategies for infections.

Proteins are also a major class of EPS components [6]. Among these, many different enzymes are involved in the synthesis and degradation of the EPS and contribute to matrix remodeling in all stages of biofilm formation. Some enzymes can produce long-chain biopolymers that are stored as nutrient source. For instance, levan, a high-molecular-mass b-(2,6)-polyfructan synthesized by the enzymes levansucrases, is stored in the voids of *Pseudomonas syringae* biofilms and used as a long-term nutrient source [55].

Enzymes are also important for biofilm detachment and dispersion when the EPS is partially degraded to release bacteria. Examples of enzymes with this function are dispersin B, which degrades N-acetylglucosamine-containing extracellular polysaccharides [48,56,57,58], and the surface protein-releasing enzyme (SPRE), that releases adhesin P1 from the bacteria surface allowing for *Streptococcus mutans* detachment from surfaces [59].

Non-enzymatic proteins play mainly a structural role by connecting the exopolysaccharide matrix with the bacteria outer membrane. For instance, lectins, a category of extracellular carbohydrate-binding proteins are implicated in cell-to-cell interactions within the biofilms, thus supporting microcolonies formation and biofilm maturation [60,61]. Biofilm-associated surface proteins, a class of high molecular mass proteins located on the bacterial surface, are known to promote biofilm formation in several bacterial species, such as *S. aureus*, by facilitating primary attachment to abiotic surfaces and intercellular adhesion [62].

Extracellular DNA (eDNA) is another important component of the EPS acting as intercellular connector and signaling molecule [16]. Its occurrence varies largely depending on the type of microorganisms present. For example, it is a major component of *P. aeruginosa* and *S. aureus* biofilms, while it is only minimally present in *S. epidermidis* biofilms [6]. Whitchurch et al. reported that treatments involving DNAses enzymes are more effective in preventing biofilm establishment rather than in disrupting mature biofilms. This indicates that eDNA plays a major role in the initial establishment of the biofilm, while its importance decreases with biofilm maturation [63]. This may be related to the increased heterogeneity of the EPS components in a mature biofilm, which makes its degradation more complex as it may require the cooperation of different enzymes [48].

Lipids may also be present in the EPS. Their role in biofilm formation and development is still unclear, but they are believed to improve adhesion on hydrophobic surfaces, particularly when combined with amphiphilic polysaccharides [6,64]. Moreover, lipidic EPS, such as surfactin, viscosin, and emulsan, may facilitate the retention of hydrophobic substances in the biofilm to support cell growth [6].

### 2.2. Mechanical Properties of Biofilms

Since the biofilm composition varies greatly depending on the microorganism species and the environmental conditions, there is also a huge variability in its mechanical properties. Moreover, biofilms are living structures, thus their properties, including the mechanical characteristics, change over time. For example, the Young’s Modulus of *P. aeruginosa* biofilms has been shown to increase from 10 Pa in the early development phase to 25 Pa when the biofilm is mature [65].

Macro-mechanical tests, such as rotational shear rheology, have given some preliminary information about the biofilm as a bulk, showing that it behaves as a viscoelastic material [66] because of its heterogeneous composition, which include water, bacterial cells, and EPS.

Viscoelastic materials present a complex shear modulus (G*) which accounts for an elastic component (represented by the storage modulus G’), and a viscous component (represented by the loss modulus G’’). When G’ > G’’ the material behaves as a viscous solid, while if G’ < G’’ the material is defined as a viscoelastic liquid. Biofilms behave as viscous solids until a yield point, where they switch to a viscoelastic liquid behavior [67]. Moreover, biofilms show shear-thinning properties since G’ decreases with increasing shear stress [68].

Environmental factors, such as shear flow, temperature, pH, and ion concentration greatly influence the biofilm mechanical properties. For instance, increasing values of the elastic modulus (from 0.9 to 100 Pa) have been reported when shear flow increases from 0.05 to 5 N/m^2^ [69,70]. *E. coli* has been shown to produce a higher concentration of curli fibers at 30 °C, resulting in a stiff biofilm with average compliance of 0.08 [71].

EPS secretion is also pH-dependent [72,73]. Group B *Streptococcus*, a bacterium involved in vaginal infections, has been shown to preferentially form biofilms at low pH. Indeed, Ho et al. evaluated the elastic moduli of biofilms grown on glass slides under different pH conditions and found Young’s Modulus values ranging from 2 to 100 kPa as the pH increased. This increase in the elastic modulus was attributed to the inability of bacteria to produce a biofilm at pH 7, since the measured value was close to that of the glass slide [74]. Moreover, the presence of multivalent cations, such as Ca^2+^, has been shown to enhance the stiffness and the stability of most biofilms [75]. For example, Körstgens et al. [76] grew bacteria under increasing calcium ions concentration (from 0.1 to 1 mg per g of agar medium) reporting an increase in the elastic modulus from 10 to 50 kPa.

Another aspect to consider is the biofilm internal heterogeneity, which results in variable mechanical properties within the matrix. Thus, evaluation of the mechanical properties at the microscale level should be performed to identify variations within the biofilm bulk [73]. For example, Galy et al. [71] used magnetic beads embedded in *E. coli* biofilm to assess the mechanical properties in different regions of the biofilm. They found three main regions: a stiff layer close to the adhesion surface, with an elastic modulus (E) greater than 200 Pa; a middle layer with E ranging between 5 and 200 Pa; and a soft external layer with E lower than 1 Pa.

It must be noted that the absence of standardized mechanical tests for biofilm characterization makes the comparisons of different studies arduous [77]. For instance, the literature reports that the apparent elastic modulus values of *P. aeruginosa* biofilms measured through shear stress technique are extremely variable, with values ranging from 0.8 to 100 Pa [69]. Similarly, the same parameter measured by compression tests ranges between 5 and 47 kPa [76]. For a more detailed discussion on this point, we suggest the interesting review by Böl et al. [66].

### 2.3. Mechanisms of Antibiotic Resistance in Bacterial Biofilms

It is known that bacterial biofilms can tolerate concentrations of antibiotics 10 to 1000 times higher than those that are harmful for their planktonic counterparts, thus increasing the possibility of chronicity of medical device-associated infections.

The mechanisms of antibiotic resistance in bacterial sessile communities have been described by Stewart and Costerton in a landmark review [78]. According to these authors, the common molecular mechanisms of antibiotic resistance do not seem to be responsible for the preservation of bacteria in a biofilm, as demonstrated by the fact that even susceptible bacteria that do not possess genes for antibiotic resistance can become highly resistant to antibiotics when they live in a sessile form. It is supposed that the remarkable resilience of bacteria in biofilms derives from the exceptional heterogeneity of cells that reside in the biofilm [78]. Microbes that reside in the inner parts of the biofilm have more difficulty in obtaining nutrients and oxygen and therefore grow slower than those living on the surface. This is a defense mechanism as many antibiotics are only active against fast growing cells, and therefore slow growing cells within the biofilm tend to be spared. Furthermore, the biofilm matrix itself can play a role in protecting the cells in the internal part of the biofilm since, besides being generally more difficult to penetrate, it is negatively charged, and this hinders the entry of positively charged antibiotics [79].

Quorum sensing (QS) is a complex system of microbial communication based on signal molecules that allows bacteria to perceive when a critical concentration of bacteria is reached and activate genes such as those for the production of virulence factors and the development of the biofilm itself [38]. In this case, QS may also be involved in antibiotics resistance development by intensifying the production of multidrug efflux pumps that expel antimicrobials from the cell.

In addition, a fascinating explanation for biofilm tolerance to antibiotics is the presence, within the biofilm, of peculiar cell types called “persisters”, i.e., slow-growing variants genetically programmed to endure environmental stress, including exposure to antibiotics [79]. When antibiotic therapy ends, persisters generate new bacterial populations leading to the recurrence of biofilm infection. Antimicrobial treatment evasion may be also related to the genetic and physiological diversity of bacterial cells living inside the biofilm that increases the possibility that some cells survive any threat [79]. These examples illustrate how well the heterogeneity among the cell types in the biofilm supports antimicrobial resistance. This heterogeneity is also related to differences in the metabolism of bacteria embedded in the biofilm.

Finally, according to other authors, the proximity of bacteria forming the biofilm enhances horizontal transfer of antibiotic resistance genes [80], making bacteria multi-resistant by spreading conventional resistance mechanisms against beta-lactam antibiotics, aminoglycosides and fluoroquinolones, such as, for example, the production of antibiotic-degrading enzymes, the generation of low-affinity antibiotic targets, and the overexpression of efflux pumps that have a wide range of substrates [38].

## 3. Biofilm Models

The last decades have seen a growing interest in the development of models to deepen the knowledge on biofilm formation and to assess the effectiveness of antibacterial treatments [25,77,81]. These models can be divided into *in vitro* and *in vivo* systems. *In vivo* models are a fundamental step to link the results of *in vitro* studies with clinical trials since they give more accurate information about the safety and the efficacy of new treatments [18]. In particular, they allow to observe the response of the host immune system to the biofilm infection [5]. However, *in vivo* models are expensive and can give rise to ethical concerns [82,83,84]. On the other hand, *in vitro* models are cheap, but allow only for preliminary assessment of treatment efficacy. They often use a single bacterial species, which is hardly ever the case in a natural environment. Moreover, they do not reproduce accurately the nutritional conditions and the substrate on which the biofilm is developed [21,85,86]. Progress has been achieved by developing 3D and microcosm models that can better replicate the biofilm architecture and the host environment [26,87,88]. However, they are still unable to fully replicate biofilm complexity.

Given the heterogeneity, complexity and evolving nature of biofilms, the design of accurate *in vitro* models is extremely challenging. Table 1 resumes the main parameters influencing biofilm formation and the effects they have on the biofilm properties. Reliable models must strive to reproduce: (i) the heterogeneous composition in terms of the different bacterial species present, (ii) the development stage, and (iii) the environmental conditions, as these parameters influence the architecture, the chemical composition, and the mechanical properties of the biofilm [5].

Each bacterium has its peculiar genotype and produces different proteins, resulting in different EPS compositions [6]. For example, *E. coli* produce a protein-based biofilm mainly made of proteinaceous curli fibres, while the main components of *Staphyloccoccus* spp. biofilm are polysaccharides [73]. Moreover, several bacterial species may be present and coexist at the same time, influencing the spatial distribution of different EPS components and therefore the final structure of the biofilm. The shape of the cells should also be considered. For instance, elongated cells such as bacillus-like bacteria are more prone to surface-adherence than coccoid cells. This results in precise spatial disposition within the biofilm, with the bacillus-like population in contact with the surface [90]. Multispecies biofilms can be more efficient in metabolizing compounds and, thus, more resistant to treatments [16]. For instance, bacteria survival in the saliva requires the synergic action of different species [89]. Moreover, in infected wounds, a combination of bacterial species is usually observed, with *S. aureus, S. epidermidis and P. aeruginosa* being the most common pathogens [9]. On the other hand, lung biofilms related to cystic fibrosis are mainly caused by a single pathogen (i.e., *P. aeruginosa*) [98]. Therefore, when developing a biofilm model, it is essential to select the relevant bacterial species (Table 2).

The stage of biofilm development is particularly important, since bacteria susceptibility to antibiotics decreases with the maturation stage [99], reducing the time window in which treatments can be effective [100]. Models that study early stages are usually easier to achieve, while reproducing biofilm dispersion is more complex since the cells should survive longer in the model.

A relevant biofilm model should mimic the surface on which the biofilm attaches and grows, the nutrient conditions and the shear stress [5]. For example, modelling infections on urinary catheters requires the inclusion of the catheter material as well as a flow of liquid simulating the urine. In this case, the most relevant phase to target is adhesion, since the treatments focus on prevention of biofilm formation [13]. On the contrary, when osteomyelitis symptoms are manifested, the biofilm is already present, and treatments focus on eradicating the infection. Therefore, a reliable osteomyelitis model requires a support matrix and a nutrient medium that simulate the bone tissue environment [87]. These models focus on obtaining mature biofilms and on studying dispersion.

The presence of specific nutrients can also activate genes that modify the morphology of biofilm and enhance its function. For instance, high iron levels induce the formation of a rugose biofilm in *E. coli* that is more resistant to disinfectants [97], while *S. epidermidis* has been shown to colonize titanium implant surfaces more efficiently depending on their roughness [29].

Moreover, results are dependent on the techniques used to detect the biofilm and to analyse and quantify its characteristic parameters. For example, several techniques are available to quantify the biofilm biomass, both physical (e.g., weighting or measuring the electrochemical impedance) and chemical (e.g., dye staining). However, the results obtained using different methods are usually difficult to compare. For instance, dye staining tends to overestimate or underestimate the biofilm biomass depending on the washing steps, while weighting is time consuming and has low accuracy [19]. Different techniques also exist for visualizing and quantifying adherent cells, which may exploit microscopy and fluorescent dyes. These techniques offer information on the spatial distribution of the cells within the biofilm and on their viability and metabolic features [101]. Particularly interesting is the exploitation of GFP-expressing or bioluminescent bacterial strains in the biofilm formation, which allows to monitor the biofilm development in real time. For instance, Sweeney et al. [102] used a luminescent *S. aureus* strain to infect bovine femur bone blocks and followed both biofilm development and bacteria metabolism over 24 h through bioluminescence imaging. Furthermore, the use of GFP-expressing or bioluminescent bacterial strains allows to spatially localize the bacteria of interest. In this regard, Wu et al. [103] recently used fluorescent strain of *S. aureus* and *P. aeruginosa* to infect a skin model and were able to observe the bacteria penetration within the skin layers. More detailed comparisons of different analysis methods can be found in the review papers by Azeredo et al. [19], Wilson et al. [101], and Merrit et al. [104].

### 3.1. 3D Models of Bacterial Biofilm

Two main approaches can be employed to obtain 3D models of biofilm *in vitro*: the static or the dynamic approach [5]. In the first case, the system is closed, and the biofilm develops on multi-well plates. The advantages of these systems are that they are cheap, do not require any specialized equipment, and allow for high throughput screening of multiple organisms and treatments [21]. However, there is no continuous nutrient replacement and waste removal, which has a deleterious effect on bacteria viability and does not recapitulate well the *in vivo* flow and nutrient conditions [105]. On the contrary, dynamic systems are open and allow the exchange of nutrients and waste, resulting also in longer lasting models. Moreover, specific environmental parameters, such as shear stress, can be considered, allowing a more reliable analysis of biofilm development and its resistance to treatments [86]. However, they are more complex and may require specific equipment and technical competences to be implemented [84].

Microcosm models are a development of static and dynamic models that aim at better replicating the biofilm environment, by including specific cells, nutrients or substrates relevant to the pathological site [5,84]. Examples of these models are described and discussed in the following paragraphs.

### 3.2. Static In Vitro Models of Biofilm

**Figure 2:**
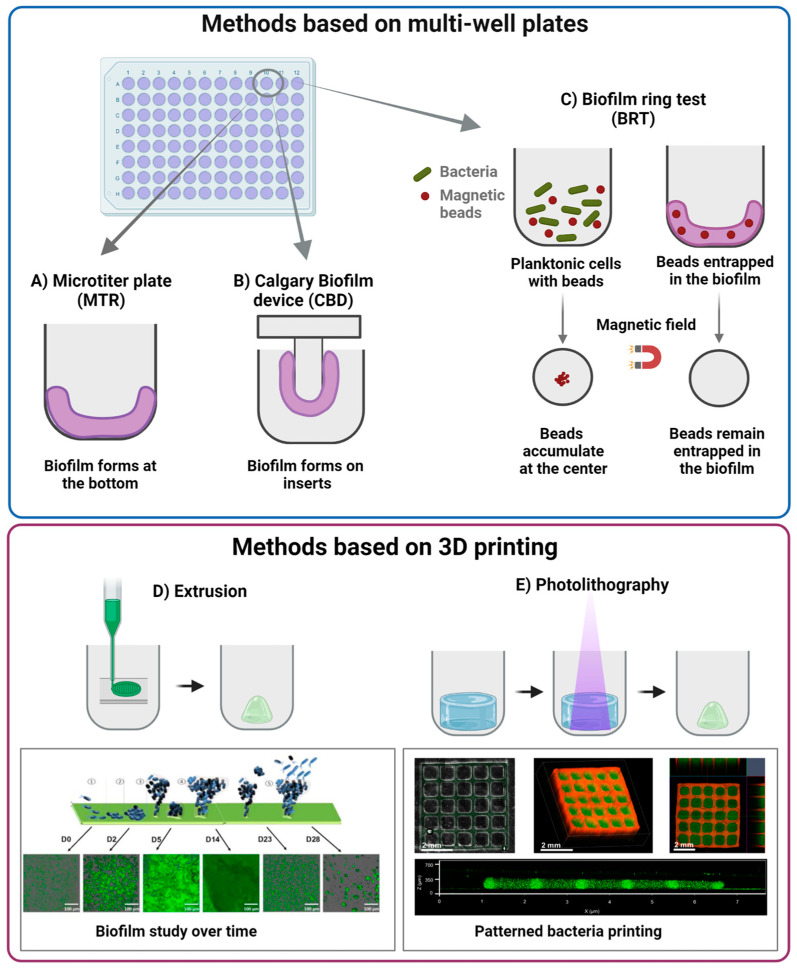
Summary of different static models. They are divided into methods based on multi-well plates: (**A**) mictrotiter plate (MTR), (**B**) Calgary biofilm method (CBD), (**C**) biofilm ring test (BRT); and methods based on 3D printing: (**D**) extrusion (inset reproduced from [22], Copyright IOP Publishing Ltd., 2019), and (**E**) photolithography (inset taken from [125], Copyright America Chemical Society, 2021). Image obtained with Biorender.

Among the static models, presented in Figure 2, the microtiter plate (MTP), the Calgary biofilm device (CBD), and the Biofilm Ring Test (BRT) are the most common.

In the MTP (Figure 2A), bacteria are grown in polystyrene wells, where they are allowed to form the biofilm. The resulting biofilm remains attached to the well bottom and can be analyzed for changes in biomass through colorimetric assays, such as crystal violet [86]. However, the method does not allow a precise quantification, since it measures also the biomass produced by sedimented cells, which are not involved in the biofilm formation.

CBD (Figure 2B) overcomes this issue by allowing the biofilm to grow on an insert attached to the coverslip. Thus, only the biomass derived from sessile development is quantified [5,19,86].

It must be noted that with crystal violet it is not possible to distinguish and quantify viable cells, since extracellular polymeric matrix and dead cells are also stained. Other methods such as the MTT assay for the assessment of cell metabolic activity and the viable cell counting method allow evaluating vital cells within the biofilm.

The MTP model can be implemented and exploited as a physical support for other types of materials used as a surface to grow microbial biofilms, such as medical-grade silicone elastomeric discs and titanium alloy discs that can be cut to fit different cell culture multi-well plates (12-, 24-, 48-, 96-well plates) [106,107,108,109,110,111]. Interestingly, these discs can be removed from the multi-well plates and analyzed by Scanning Electron Microscopy (SEM) for microstructural characterization of microbial biofilms [107,108,112]. The use of discs or coupons also allows the washing of the biofilms and the removal of non-adherent and sedimented cells. In this way, it is possible to prolong the life of the biofilm by moving the disks into wells containing fresh medium, providing it with new nutrients to continue the growth [107,113,114].

In addition, this approach has been successfully used to cultivate multi-species biofilms such as the inter-kingdom *Candida albicans - S. aureus* and *C. albicans - S. epidermidis* model biofilms on different materials as silicone, titanium, glass, stainless steel, polystyrene [107,108,114,115,116,117]. An accurate setup of the experimental procedures such as the optimization of microbial inoculum concentration, the selection of the appropriate culture media (for inter-kingdom biofilms generally a 1:1 mixture of bacterial and yeast media as yeast extract-peptone-dextrose (YPD) and tryptone soy broth (TSB) or simply Roswell Park Memorial Institute Medium (RPMI) supplemented with 2% glucose) and appropriate cultivation conditions (e.g., biofilm washing and replenishment of fresh culture media) support continuous *in vitro* growth of mixed biofilms. Inter-kingdom polymicrobial biofilms composed by *Staphylococci* and *Candida* are difficult to diagnose and constitute a severe risk of chronic systemic infections due to the absence of any common therapeutic target for their eradication [114]; they generally necessitate complex multi-drug therapy and, in most cases, the removal of contaminated medical devices. These models are, thus, of fundamental importance to evaluate the effectiveness of multiple and innovative treatment approaches.

Moreover, both MTP and BCD can be improved by coating the wells with different substrates to better mimic the stiffness and the chemical properties of the surface the biofilm adheres to. Such coatings can be made of natural polymers, such as collagen [118], hyaluronic acid [26,119] and fatty acids [88], or synthetic polymers, such as Poloxamer® [120] and poly(ethylene glycol) [121]. An interesting example is the model of Grassi et al. to test innovative treatments [26,119] in which an artificial infected dermis composed of two layers of hyaluronic acid and collagen simulates the chronic wound environment. However, these techniques do not allow to follow biofilm formation precisely and can only be used to mimic the later phases of biofilm development and maturation [19].

BRT (Figure 2C) is based on the capacity of bacteria to entrap magnetic beads during early phases of biofilm formation. When a magnetic field is applied, free magnetic beads move toward the magnetic field and the level of biofilm maturity can be assessed by the quantity of entrapped beads. This can be performed at different timepoints to study also the early phases of biofilm development [19,86]. The main common limitation of the above methods is that the biofilm usually develops on a 2D substrate which does not recapitulate the 3D complexity of the *in vivo* environment, resulting in biofilms that do not replicate bacteria resistance [22].

To better reproduce the biofilm architecture and its mechanical properties, 3D-printing techniques have been recently proposed with promising results, as summarized in Table 3. These techniques use bioinks (i.e., cell-loaded polymeric solutions that can be printed and form a gel following an external stimulation). The bioink constituent polymers can be either of natural or synthetic origin. Among natural polymers, alginate, gelatin, and hyaluronic acid are widely used by virtue of their biocompatibility [22,122,123,124], while poly(ethylene glycol) is one of the most used synthetic polymers due to its versatility and tunability [125]. Bioinks are optimized to embed vital cells that could be printed with high precision [126,127]. For instance, several research groups [22,123,124] obtained stable biofilm models from different bacterial species by extrusion 3D printing. Such models were maintained in culture for more than a week and allowed to control the bacterial species present, their spatial disposition, and their density. For instance, Ning et al. [22] developed an alginate-based bioink to print three different bacterial species, *E. coli, P. aeruginosa* and *S. aureus*. The alginate-bacteria solution was initially crosslinked by exposure to calcium chloride to obtain a hydrogel sufficiently viscous to be printed, followed by a second crosslinking with barium chloride that increased the biofilm stability to over 28 days. The biofilm life cycle was studied from early colonization to dispersion by analyzing cell viability at different time points (Figure 2D). However, the thickness of the biofilm obtained was higher than what is found in natural environment, due to intrinsic resolution limitations of the printing technique. Photolithography has higher printing resolution, thus allowing a better replication of the biofilm complexity [122,125]. Dubbin et al. [125] were able to pattern distinct *E. coli* strains within defined geometries (Figure 2E). They also showed that bioinks with different storage modulus values could be printed to mimic matrices of different stiffness. They reported that less rigid materials allowed the formation of larger microcolonies. However, the model was stable for only 3 days, limiting the possibility to study mature biofilms. Moreover, photolithography is an expensive technique and is not suitable for high-throughput analyses [122].

### 3.3. Dynamic In Vitro Models of Biofilm

**Figure 3:**
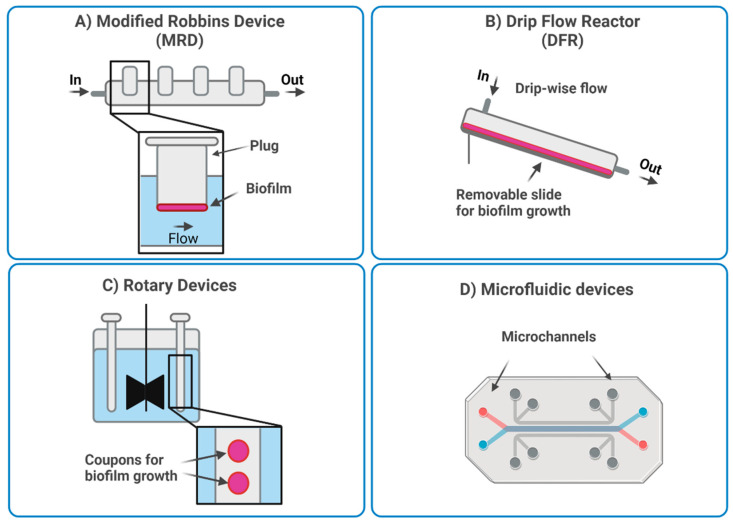
Summary of different dynamic models: (**A**) modified Robbins device (MRD), (**B**) drip flow reactor (DFR), (**C**) rotary devices, and (**D**) microfluidic devices. Image obtained with Biorender.

Dynamic systems allow a continuous flow of nutrients and waste products. Thus, biofilm development can be studied over several weeks. The most employed dynamic models, shown in Figure 3, are the modified Robbins device (MRD), the Drip flow reactor (DFR), rotary reactors, and microfluidic systems [19,21,86]. In the MRD and DFR (Figure 3A and Figure 3B, respectively), the biofilm grows under selected hydrodynamic conditions (e.g., flow rate, shear stress) on coupons or microscopy slides that can be removed for analysis. The MRD systems are composed of a pipe with holes in which the coupons are placed. The flow speed inside the pipe can be controlled so that biofilm development can be studied under different flow conditions. For instance, Raad et al. studied the effect of different antimicrobials in a model of infected catheter developed within a MRD system [128]. To produce the model, silicone catheter segments were placed on the specimen plugs of the MRD system and maintained in contact with the solution containing two different microbial species involved in catheters infections (i.e., methicillin-resistant *S. aureus* (MRSA) and *Candida parapsilosis*). The obtained infected catheters were then incubated with different antimicrobials to assess their effectiveness. In another study, Blanc et al. [129] used MRD to reproduce the oral environment, by mimicking the flow of saliva. The authors achieved the formation of a six-species biofilm on hydroxyapatite disks and observed spatial heterogeneity within the biofilm, with higher viability of the bacteria closer to the biofilm surface. 

DRF systems consist of a tilted chamber in which a slide is inserted. During operation, a small amount of fluid passes through the chamber, producing slow shear conditions. This system is suitable for studying the heterogeneity and presence of gradients within the biofilm [130]. For example, Xu et al. [131] studied the impact of oxygen availability on the heterogeneity of *P. aeruginosa* biofilm by imaging the alkaline phosphatase (APase) activity inside the biofilm, following exposure to a fluorescent substrate. They showed that cells in the upper layers had a higher APase activity and were more metabolically active, due to easier access to oxygen. 

As shown in Figure 3C, rotatory reactors are composed of rotating cylinders or disks that provide surface shear. The coupons can be placed either on the rotating or on the fixed structure and are subjected to shear stress depending on their angular positioning. The advantage of these systems is that shear stress is given by the disk rotation and not by the flow rate, therefore these two parameters can be controlled independently [19,21]. An example of rotary reactors is the Centre for Disease Control (CDC) biofilm reactor in which several rods containing coupons are placed in a vessel and the shear stress is provided by magnetic stirring in the center. In this way, biofilms can be grown under shear stress, as compared to MRD systems [86]. The CDC biofilm reactor is routinary used in the standard methods (E2196-12 and E2562-12) of the American Society for Testing and Materials (ASTM) to assess biofilm formation and prevention on surfaces and devices [19,132]. For example, it has been used to study the efficacy of antibacterial treatments against *S. aureus* and *P. aeruginosa* biofilms on implantable cardiac devices [132,133]. 

In general, the limitations of dynamic systems are that they are more complex than static systems and often require more specialized equipment. Moreover, only a few bacterial species or a few combinations of species can be tested during the same experiment, due to the absence of isolated chambers [19,86]. To solve this issue, an innovative dynamic system was proposed by Duckworth et al. in 2018 [134] to model chronic wound infections. In this model, the biofilm grew in a well on a semi-permeable membrane, and it was fed from beneath through a channel connected to a pump. This allowed for the simultaneous culture of different bacterial species without the risk of cross contaminations (Figure 3D). Moreover, the model allowed to control additional parameters such as relative abundance of the bacterial species, growth time, temperature, nutrient type, and nutrient supply rate. The model was used to study the development of a two-species biofilm formed by *S. aureus* and *P. aeruginosa* over 72 h. The authors observed that the prevalence of the species changed over time with *S. aureus* being predominant in the first 10 h and *P. aeruginosa* becoming most numerous after 10 h. This was in accordance with what was reported in patients with infected chronic wounds. Moreover, the model was applied to the testing of antimicrobial dressings, observing a lower efficacy than what found with traditional static models. This was related to the higher similarity of the model to the *in vivo* environment. Moreover, results were highly reproducible, confirming the robustness of the model.

Microfluidic systems are composed of *ad hoc* designed microchannels that allow fine-tuning of several parameters, such as nutrients, signal molecule levels, and flow conditions. Moreover, microfluidic systems are compatible with different detection methods, either off-chip or on-chip. For example, samples can be collected from the chambers and analyzed, while optical microscopy can be used for real time monitoring on the microfluidic device [19]. 

Microfluidic systems can be expensive and complicated to handle. On the other hand, they offer the advantage of controlling parameters at the single cell level resulting in a more accurate study of biofilm [19]. For example, gradients can be established for the study of bacteria within biofilms, or to expose bacteria and biofilms to new treatments [24,135]. Lee et al. [24] developed a microfluidic system to study biofilm dispersion induced by Dispersin B. They were able to control Dispersin B delivery and to combine it with antibiotics, observing bacteria release from the preformed biofilm. Moreover, the imaging of the microfluidic channel showed the successful removal of the biofilm after the treatment. Because of their fine tunability, microfluidic systems can be used to recreate more precisely the conditions found in the biological environment. For example, Wright et al. [135] developed a microfluidic model to reproduce wound infections. They produced gradients inside the microfluidic system to mimic the discontinuous availability of nutrients and they studied the effect on bacteria chemotaxis. They observed changes of motility according to the nutrient gradient, supporting the notion that nutrient availability facilitates the transition from planktonic to sessile state.

Microfluidic systems also allow the development of biofilm in conditions that are difficult to achieve with traditional dynamic methods. For example, Jung et al. [23] developed a microfluidic device in which a biofilm was obtained in the absence of shear stress. Using a V-shaped channel, they were able to supply culture medium to a chamber containing agarose-embedded bacteria without producing shear forces. The authors were able to obtain a biofilm not attached to a surface but rather embedded in a matrix and they could image the stages of its development. This condition simulates clinical biofilms forming in mucosa layers or in the intracellular space.

### 3.4. Microcosm Models

Microcosm models can be static or dynamic systems which include additional features, such as cells, materials, or nutrients to better mimic the pathological environment [5,136,137].

To reproduce the pathological/physiological environment in which the biofilm develops, microcosm models can include artificial or *ex vivo*-derived substrates, cells, and media to simulate a disease-specific ECM. They can also include more than a single bacterial species to model biofilm complexity. Moreover, they should be designed for long term study by including systems for continuous media recycling to extend the lifespan of the model. Examples of microcosm models and their main features are presented below.

For instance, Raic et al. [87] developed an osteomyelitis model by culturing *P. aeruginosa* or *S. aureus* on a 3D bone marrow analogue composed of human hematopoietic stem and progenitor cells (hHSPCs) seeded on an artificial scaffold. With this model they could follow proliferation and differentiation of hHSPCs over 7 days and study the effects of infection on hematopoiesis. Similarly, Grassi et al., Carterson et al. and Crabbé et al. [26,138,139] developed a model of cystic fibrosis by seeding human adenocarcinoma alveolar epithelial cells on type I collagen-coated dextran beads and infected the alveolar model with *P. aeruginosa*. They could confirm through microscopy the formation of biofilm on the alveolar model without impairing cell viability and adhesion to the beads. Moreover, the biofilms grown in association with epithelial cells were more resistant to antibiotic treatment, thus better mirroring the *in vivo* situation.

*Ex vivo*-derived tissues can also be used to develop microcosm models. Sánchez et al. [137] studied the formation of multi-species biofilm on implant surfaces, by using saliva collected from donors to coat the implants prior to bacteria seeding to better simulate the oral environment. They were able to follow and characterize the development of the biofilm until maturation in terms of structure and bacterial composition, demonstrating consistency with *in vivo* observations. Similarly, Liu et al. [140] inoculated human enamel blocks with homogenized plaques collected from human donors to simulate the environment in which dental caries develop. Through confocal topography imaging and transversal microradiography analysis, they were able to observe the presence of micro-cavities similar to the early caries lesions on the surface of infected enamel blocks. Wang et al. [141] also developed a model for studying caries, by using human teeth from donors. Then, after different antibacterial treatments, they used scanning electron microscopy to observe the morphology of the bacteria on the surface and evaluate the effects of the treatments. Besser et al. [142] developed an infected would model by using plasma containing also the immune competent cells from the buffy coat from donors. They mixed the blood-derived tissue with *S. aureus* or *P. aeruginosa* and induced fibrin polymerization. They observed the deposition of EPS and the interaction between leucocytes from the buffy coat and pathogens. They also reported an increased biocide tolerance with biofilm maturation, as experienced in clinical conditions, confirming the relevance of the system.

In some cases, artificial and *ex vivo*-derived materials can be combined to obtain a model. Zhou et al. developed a dental plaque biofilm model [143] to test a new resin composite to be applied for restoring root caries that could also prevent biofilm formation and promote remineralization. To prove the efficacy of this composite, the authors extracted dentin slabs from bovine teeth, combined them with the resin composite and cultured the obtained material with a multi-species inoculum over a period of 7 days. Interestingly, in this work *S. mutans* and *L. acidophilus* were combined with *C. albicans* to form an inter-kingdom biofilm. This is a particularly relevant aspect since multi-species biofilms are typical of oral cavity diseases.

Another important aspect in biofilm studies is the long-term assessment of the model. This is particularly challenging when the model contains human cells that could have their viability impaired by the presence of bacteria. Indeed, the models developed by Besser et al. [142] and Crabbé et al. [139], which contained human cells in co-culture with bacteria had a limited lifespan of 72 h and 17 h, respectively. On the contrary, models with only bacterial cells could be maintained for at least 7 days.

Although microcosm models represent a promising approach to reproduce biofilm growth and development, further research is needed to improve the complexity and to extend the lifespan of these modes to achieve better mimicry of the pathological conditions.

## 4. Discussion and Conclusions

Bacterial infections and the development of antibiotic resistance, which are strictly related to the capacity of bacteria to organize in complex biofilms, are a growing concern for the health care system.

In the last decades, several studies aimed at disclosing biofilm architecture, mechanical properties, and chemical structure, revealed the extreme complexity and heterogeneity of such systems. These differences are mainly related to the presence of multiple bacterial species, and to the environment in which the biofilm grows.

As a result, new *in vitro* models have emerged as useful tools to replicate the biofilm environment and its complexity.

In this review, the main *in vitro* models were described, and their advantages, disadvantages, and possible applications were discussed.

Static models are usually easier to handle and can include scaffolds made of natural or synthetic polymers to better imitate *in vivo* substrates or stimulate the EPS. Furthermore, 3D printing represents an emerging approach for the design of static biofilm models. However, the available reports focus mainly on technical/design aspects of the models, such as ink formulation and printing setup, with less attention on model interrogation and exploitation. An intrinsic problem of static models is that they do not allow for continuous renew of nutrients and disposal of waste products, which reduce the model lifespan. Moreover, the absence of shear stress reduces the range of applications for these models.

On the contrary, dynamic models are more versatile and relevant, since they allow the control of important parameters, such as nutrient flow and shear stress. Of particular interest are microfluidic devices, which allow to set specific parameters, such as nutrient and drug concentration, and to control them at the single cell level. However, these systems are expensive, usually custom-made, and require specific training to be handled. Moreover, they are not always optimal to mimic the *in vivo* environment of pathologies that do not present shear forces (e.g., osteomyelitis or infections on bone fixation devices).

A final approach analysed in this review is the microcosm model. These models recreate the environment in which the biofilm develops by including cells, scaffolds, or materials from donors, thus being more clinically relevant. However, the comparison performed showed that the models developed so far are still unable to recapitulate all the aspects of *in vivo* complexity. Multi-species, and inter-kingdom models are still very rare and their stability in the long term is still an issue.

Further studies are required to increase our understanding of biofilms and bacteria interactions and to develop and refine the available techniques for producing biofilm models, to achieve relevant tools for drug testing/screening and refine the use of *in vivo* models.

## Figures and Tables

**Table 1:** Parameters influencing biofilm formation with regard to bacterial metabolism, EPS production and mechanical properties.

Parameter	Effects on Bacterial Metabolism	Effects on EPS Composition	Effects on Biofilm Mechanical Properties	REF
Bacterial species	Different strains have different metabolic activity	Bacteria-specific EPS Quantity of secreted EPS	Biofilms produced by different bacterial species have different mechanical properties	[16,66,73]
Multispecies	Bacteria cooperate in production and metabolization of nutrients	Secretion of different EPS components	Cell shape influences bacteria disposition and, thus, biofilm architecture	[16,89,90]
Temperature	Affects cell viability and metabolism	Quantity and composition of EPS	Affects biofilm stiffness	[71,91,92]
pH	Alters gene expression	Quantity and composition of secreted EPS	Bacteria grown under optimal pH conditions produce stiffer biofilms	[72,74,93,94]
Shear flow	Stimulates bacteria detachment	-	Higher shear flow results in higher stiffness	[69,70,95,96]
Nutrients and oxygen	Development of different bacteria phenotypes	Bacteria-specific EPS	Biofilm produced by different bacterial species have different mechanical properties.	[5,51]
Ion concentration	Alters gene expression	Quantity and composition of secreted EPS	Multivalent cations enhance stiffness	[75,76,97]

**Table 2:** Characteristics of biofilms in common infections, considering the main bacterial species involved and the phases of biofilm development more important to study the pathological situation.

Tissue	Application	Main Bacterial Species Involved [9]	Phase of Biofilm Development
Skin	Wound healing	*S. aureus* *S. epidermidis* *P. aeruginosa*	Adhesion Dispersion
	Acne	*Propionibacterium acnes* *Propionibacterium cellulitis* *Propionibacterium erysipelas.*	Adhesion Dispersion
Bone	Osteomyelitis	*S. aureus* *S. epidermidis*	Dispersion
	Dental plaques	*Streptococcus mutans* *Lactobacillus* spp. *Actinomyces* spp.	Adhesion
Lung	Cystic fibrosis	*P. aeruginosa*	Adhesion Dispersion
Implanted devices and catheters	Implant infection	*S. aureus* *S. epidermidis* *Enterococcus faecalis* *E. coli* *Klebsiella pneumoniae* *Proteus mirabilis* *P. aeruginosa*	Adhesion

**Table 3:** Comparison of different 3D-printed models.

Bacteria	Bioink	3D-Printing Method	Pros	Cons	REF
*P. putida*, *A. xylinum*	Hyaluronic acid k-carrageenan fumed silica	Extrusion	Multispecies biofilm. Bacteria produced cellulose to replace the bioink.	No characterization on the biofilm stage.	[123]
*E. coli*	Alginate	Extrusion	Stability >1 week. Bacteria produced their own EPS to replace alginate.	Use of genetically modified bacteria that secrete EPS following external stimulation.	[124]
*E. coli*, *P. aeruginosa*	Alginate	Extrusion	Stability >4 weeks. Suitable for aerobic and anaerobic bacteria.	Minimum thickness of 0.25 mm (natural biofilm can be <0.1 mm).	[22]
*S. aureus*, *P. aeruginosa*	Gelatin—A	Photolithography	Multispecies biofilm. Study of cell-cell communication.	Expensive. Low-throughput analyses.	[122]
*E. coli*	PEG diacrylate (PEGDa)	Photolithography	High resolution (10 µm). Complex geometries. Modulation of the mechanical properties of the bioink.	Low stability (3 days).	[125]

## Data Availability

No data were created.
